# Functional diversity and properties of multiple xylanases from *Penicillium oxalicum* GZ-2

**DOI:** 10.1038/srep12631

**Published:** 2015-07-30

**Authors:** Hanpeng Liao, Haiping Zheng, Shuixian Li, Zhong Wei, Xinlan Mei, Hongyu Ma, Qirong Shen, Yangchun Xu

**Affiliations:** 1 National Engineering Research Center for Organic-based Fertilizers, Jiangsu Collaborative Innovation Center for Solid Organic Waste Utilization, Nanjing Agricultural University, Nanjing, 210095, China; 2 Ningbo Urban Environment Observation and Research Station-NUEORS, Institute of Urban Environment, Chinese Academy of Sciences, No. 88 Zhong Ke Road, Ningbo 315830, China; 3 College of plant protection, Nanjing Agricultural University, Nanjing, 210095, China

## Abstract

A multiple xylanase system with high levels of xylanase activity produced from *Penicillium oxalicum* GZ-2 using agricultural waste as a substrate has been previously reported. However, the eco-physiological properties and origin of the multiplicity of xylanases remain unclear. In the present study, eight active bands were detected using zymography, and all bands were identified as putative xylanases using MALDI-TOF-MS/MS. These putative xylanases are encoded by six different xylanase genes. To evaluate the functions and eco-physiological properties of xylanase genes, *xyn10A*, *xyn11A*, *xyn10B* and *xyn11B* were expressed in *Pichia pastoris*. The recombinant enzymes xyn10A and xyn10B belong to the glycoside hydrolase (GH) family 10 xylanases, while xyn11A and xyn11B belong to GH11 xylanases. Biochemical analysis of the recombinant proteins revealed that all enzymes exhibited xylanase activity against xylans but with different substrate specificities, properties and kinetic parameters. These results demonstrated that the production of multiple xylanases in *P. oxalicum* GZ-2 was attributed to the genetic redundancy of xylanases and the post-translational modifications, providing insight into a more diverse xylanase system for the efficient degradation of complex hemicelluloses.

Xylans, the major component of hemicellulose, are composed of a backbone of β-1,4-linked D-xylopyranosyl residues and side chains containing different substituents^1^. The complete breakdown of xylans requires a variety of hydrolytic enzymes, such as xylanase (EC 3.2.1.8), β-xylosidase (EC 3.2.1.37), α-L-arabinofuranosidase (EC 3.2.1.55), α-D-glucuronidase (EC 3.2.1.139), and acetyl xylan esterase (EC 3.1.1.72)^2^. Among these enzymes, xylanase is an important enzyme for the cleavage of internal β-(1,4)-linked D-xylosyl glycosidic bonds in heteroxylan to generate short xylo-oligosaccharides^3^. Most reported xylanases are classified into glycoside hydrolase (GH) families 10 and 11 based on amino acid similarities, whereas a minority of these enzymes belong to GH30, GH7, GH8, and GH43 families (http://www.cazy.org). The potential application of xylanases in numerous biotechnological processes, such as the bleaching of pulp and bioconversion of lignocellulosic materials and agro-wastes into fermentative and animal feed/food products for textile and fuel industries, respectively, has been proposed^3^,^4^.

Numerous filamentous fungi exhibit high xylan degradation, as these microbes possess multiple xylanases from various families^5^,^6^. *Aspergillus ochraceus* produces at least three independent xylanases^7^, *Trichoderma reesei* produces six independent xylanases^8^, and the xylanolytic fungus *Talaromyces versatilis* produces four independent GH11 xylanases with various properties^9^. Plant xylans have a complex structure and heterogeneous composition^4^, and not all of the xylosidic linkages in heteroxylans are equally accessible to xylanases^10^. Therefore, multiple xylanases with diverse biochemical properties, structures, specific activities, and substrate specificities are required to increase the efficiency and extent of xylan hydrolysis^11^,^12^. Currently, hundreds of xylanases have been widely reported and characterized^3^,^8^,^13^,^14^,^15^,^16^,^17^, but few studies have focused on the multiplicity of functions and eco-physiological properties of these enzymes. Particularly, there are few reports on the occurrence of multiple xylanases from *Penicillium*.

In previous studies using agricultural residue as a substrate, we observed that the fungus *P. oxalicum* GZ-2 produces multiple xylanases with high levels of enzymatic activity^5^,^18^. However, several questions remain regarding the role and origin of the multiple xylanases identified in strain GZ-2 and the relationships of each independent xylanase; differences in the xylanase diversity between microbes; and whether these enzymes are encoded by redundant genes. Here, we investigated the origin of multiple xylanases and their functions and biochemical properties in *P. oxalicum* GZ-2 based on genetic data and biochemical determinations.

## Results

### Detection and identification of multiple xylanases from *P. oxalicum* GZ-2

**Figure 1:**
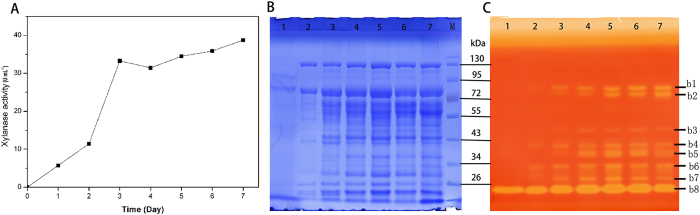
The production of multiple xylanase enzymes was evaluated by different assay methods. (**A**) Xylanase activity was determined by a DNS method during *P. oxalicum* GZ-2 incubation at 30°C for 7 days induced by corn cob. (**B**) SDS-PAGE analysis of culture filtrates on various culture days. (**C**) Zymogram analysis of culture filtrates on various culture days. Lane 1 – 7: culture supernatants after induction for 1, 2, 3, 4, 5, 6, and 7 days; Lane M: protein molecular mass makers.

The production of multiple xylanases in *P. oxalicum* GZ-2 was evaluated using DNS assays and zymography, and the results are shown in Fig. 1. The xylanase activity of the culture supernatant gradually increased with time, reaching the highest value at the end of fermentation when *P. oxalicum* GZ-2 was grown on corncob (Fig. 1A). The protein profiles of the culture supernatant of the induction period from 2 to 7 days showed that many proteins are induced by agricultural waste, and these enzymes were further analyzed through SDS-PAGE (Fig. 1B). Zymogram analysis of the culture supernatants revealed multiple xylanase protein bands (8 bands) (Fig. 1C), corresponding to a high level of xylanase activity in the culture supernatant after day 3 of fermentation. On the first day of fermentation, only one low-molecular-weight xylanase was secreted. With increased fermentation time, the number of xylanase active bands was increased. These active bands were excised, trypsin digested and further identified using MALDI-TOF-MS/MS. The results shown in Table 1 revealed six genetically different xylanases belonging to GH10, GH11 and GH30 families and two xylanase proteins with the same genotype and different molecular weights.

### Cloning and sequence analysis of xylanase genes from *P. oxalicum* GZ-2

**Figure 2:**
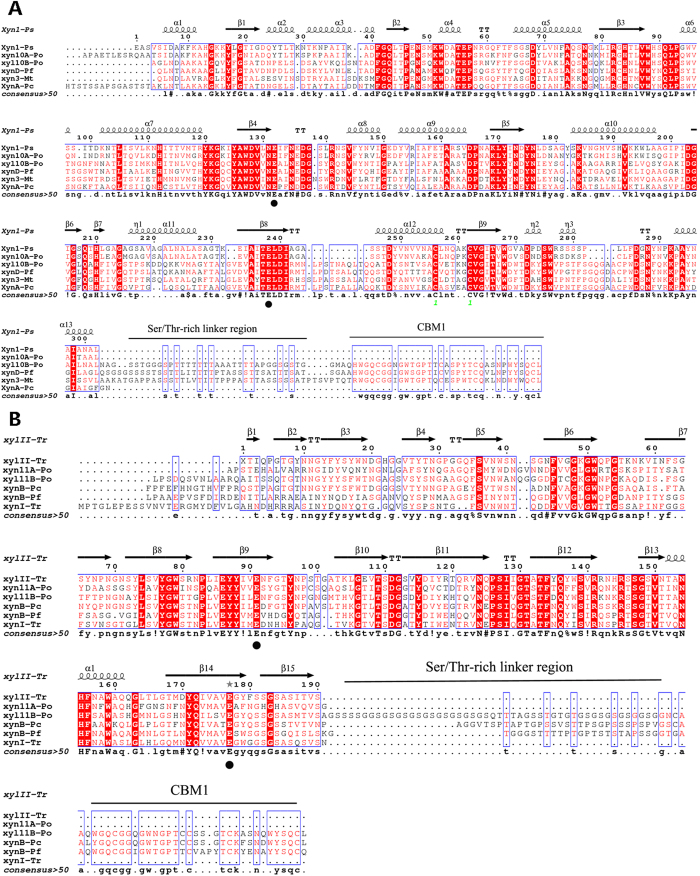
Amino acid sequence alignment of endo-β-1 4-xylanase from *P. oxalicum* GZ-2 with related fungi. (**A**) The secondary structural elements (α-helices are displayed as squiggles, β-strands are rendered as arrows, and strict β-turns are represented by the letters TT) of xyn10A and xyn10B using *Penicillium simplicissimum* xylanase as a template (pdb no. 1B3Z). The alignment includes xyn10A (KF233755) and xyn10B (KF233757) from *P. oxalicum* GZ-2, XynA from *Phanerochaete chrysosporium* (AAG44993.1), xyn3 from *Myceliophthora thermophila* (JF508854), and xynD from *Penicillium funiculosum* (CAG25554). (**B**) The secondary structural elements of xyn11A and xyn11B using *Trichoderma reesei* xylanase as a template (pdb no.1ENX). The alignment includes xyn11A (KF233756) and xyn11B (KF233758) from *P. oxalicum* GZ-2, xynB from *Phanerochaete chrysosporium* (AAG44994), xynB from *Penicillium funiculosum* (CAD33900), and xynI from *Trichoderma reesei* (P36218). Strictly conserved residues are highlighted with a red background and conservatively substituted residues are boxed. The conserved catalytic residues are indicated by a black dot. The figure was constructed with ESPript 3.0.

**Figure 3:**
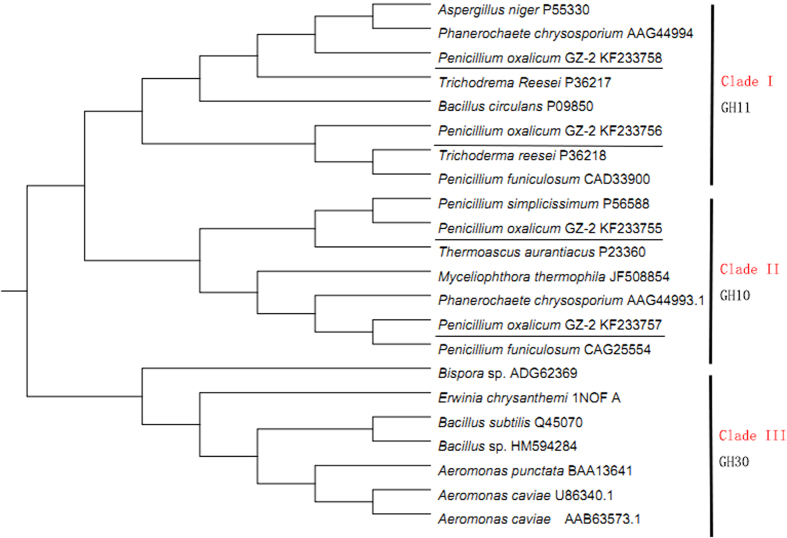
The phylogenetic tree resulting from the analysis of *P. oxalicum* GZ-2 xylanases and other xylanase amino acid sequences using the Neighbor-Joining method. The numbers on nodes correspond to the percentage bootstrap values for 1,000 replicates.

Three gene fragments (*xyn10A*, *xyn10B* and *xyn11B*) were amplified from the genomic DNA of strain GZ-2 using the corresponding degenerate primers (Table S1, supplementary). Using SEFA-PCR, complete fragments were assembled based on the core regions to create a sequence containing a complete chromosomal gene predicted using FGENESH (http://linux1.softberry.com/berry). The BLAST result indicated that the predicted sequences show high similarity to xylanase. The nucleotide sequence analysis showed that the sequences of *xyn10A*, *xyn10B* and *xyn11B* contain open reading frames (ORF) of 993, 1233, and 933 bp, respectively. Seven, four and two introns were detected in *xyn10A*, *xyn10B* and *xyn11* genes, respectively, according to the alignment of the DNA and cDNA sequences. The presence of 19, 22, and 19 amino acid signal peptides at the N-terminus were predicted using the SignalP server system for xyn10A, xyn10B and xyn11B, respectively (http://www.cbs.dtu.dk/services/SignalP/), indicating that these proteins are extracellular enzymes. The deduced mature proteins of xyn10A, xyn10B, and xyn11B contained 312, 389, and 292 residues, respectively. Molecular masses of 34.2, 41.4, and 29.5 kDa and theoretical pI values of 8.2, 5.9, and 6.5 were calculated for mature xyn10A, xyn10B and xyn11B, respectively (http://web.expasy.org/compute_pi/). Based on the analysis of the deduced amino acid sequence using the BLAST tool, xyn10A contains a catalytic domain typical of GH10 xylanases and no carbohydrate-binding domain; in contrast, GH10 xyn10B and GH11 xyn11B not only contain a catalytic domain but also have a Ser/Thr-rich linker region and a carbohydrate-binding domain with significant similarity to family 1 carbohydrate-binding modules (http://pfam.sanger.ac.uk/search/sequence) (Fig. 2). Conserved domain searches and amino acid sequence alignments of the four xylanases with other GH10 and GH11 xylanases revealed a conserved active site for GH10 glycoside hydrolases in both the xyn10A and xyn10B and regions of the active site of GH11 glycoside hydrolases in xyn11A and xyn11B (Fig. 2). The conserved domain alignments also predicted that xyn10B and xyn11B included a family 1 carbohydrate-binding module (CBM1), formerly referred to as a type I fungal cellulose-binding domain^19^. These alignments also revealed that the Glu140, Glu246, Glu130, and Glu237 catalytic domain residues were highly conserved among the six GH10 and GH11 fungal xylanases (Fig. 2). A phylogenetic tree generated from 22 candidate sequences revealed the evolutionary relationship among multiple xylanases using the Neighbor-Joining (NJ) method. The results showed three evolutionarily related clades, and each clade represented one family of glycoside hydrolases (Fig. 3). Clade I, Clade II and Clade III represent GH11, GH10 and GH30 xylanases, respectively. As shown in the phylogenetic tree, Clade I (or Clade II) consists of two subclades, including xyl10A and xyl10B (or xyl11A and xyl11B).

### Heterologous expression and purification of four xylanases

**Figure 4:**
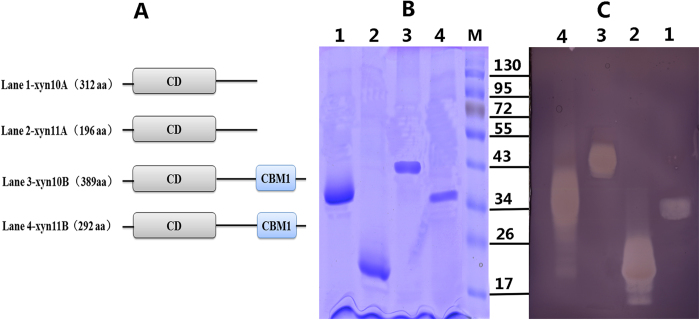
Comparative analysis of the structure and molecular weight of four xylanases. (**A**) The amino acid structure of four xylanases. CD, catalytic domain; CBM1, carbohydrate binding module from family 1. (**B**) SDS-PAGE analysis of four purified recombinant xylanases. (**C**) Zymogram analysis of four purified recombinant xylanases. Lane M: protein molecular mass makers.

To examine the biochemical functions and properties of the xylanases from *P. oxalicum* GZ-2, the cDNA of xyn10A, xyn11A, xyn10B, and xyn11B was cloned into the pPICZαA plasmid and expressed in *P. pastoris* as a recombinant protein using the downstream native α-factor signal peptide sequence under the control of the AOX1 promoter. The enzymes xyn10B and xyn11B both contained catalytic and carbohydrate-binding domains at the N-terminus belonging to the CBM1 family (Fig. 4A). In contrast, the carbohydrate-binding domain was absent in both xyn10A and xyn11A. The Ser/Thr-rich linker region and carbohydrate-binding domain showed significant similarity to other known fungal xylanases. The resulting plasmid containing the target genes of xyn10A, xyn11A, xyn10B, and xyn11B was subsequently transformed into *P. pastoris* through electroporation. After induction of protein expression in *P. pastoris* using methanol, the xylanase activities were detected in the culture filtrate of yeasts carrying the recombinant target genes but were not present in the supernatant of untransformed cells (data not shown). Because all expressed proteins contained 6×His-tags, all recombinant xylanases were purified to electrophoretic homogeneity through Ni-NTA affinity chromatography. SDS-PAGE revealed that the purified recombinant proteins of xyn10A, xyn11A, xyn10B, and xyn11B showed single bands with apparent molecular masses of 36, 23, 43, and 32 kDa, respectively (Fig. 4B). The zymogram also demonstrated that all purified recombinant proteins of xyn10A, xyn11A, xyn10B, and xyn11B exhibited xylan degradation (Fig. 4C).

### Comparison of the expression profiles of four xylanase genes induced through various substrates

**Figure 5:**
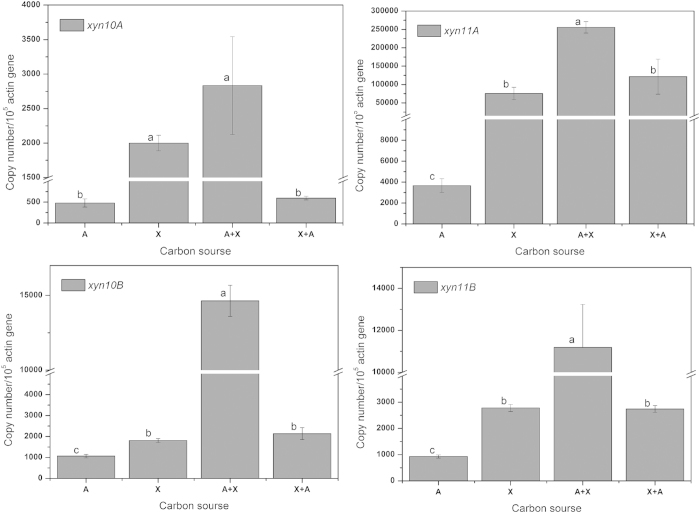
Four xylanase gene expression levels in *P. oxalicum* GZ-2 grown on various substrates. (**A**) Avicel, X: xylan, A+X: addition of X to A culture medium, X+A: addition of A to X culture medium.

To examine the expression profiles of the multiple xylanolytic system of *P. oxalicum* GZ-2, we determined the expression of four xylanase genes induced using various substrates. These xylanase genes were constitutively expressed in all substrate treatments (Fig. 5). The transcript level of *xyn11A* was significantly higher (*P *< 0.05) than that of the other xylanase genes. The most suitable treatment to induce the expression of multiple xylanases among the four tested treatments was A+X. Interestingly, the four xylanase genes were expressed when A was used as the sole carbon source for *P. oxalicum* GZ-2. The expression levels of the four xylanase genes induced by X were significantly higher than those induced by A. Surprisingly, significantly higher transcript levels were detected for all xylanase genes when X was added to the A culture than when cells were treated with no X supplement. A comparison of the transcript levels between X and X+A showed that adding A to the X culture did not affect *xyn11A*, *xyn10B* and *xyn11B* gene expression. In contrast, the transcript level of *xyn10A* was significantly decreased when A was added to the X culture.

### Comparison of the biochemical parameters of the four recombinant xylanases

#### Effect of temperature and pH on enzyme activity and their stability

**Figure 6:**
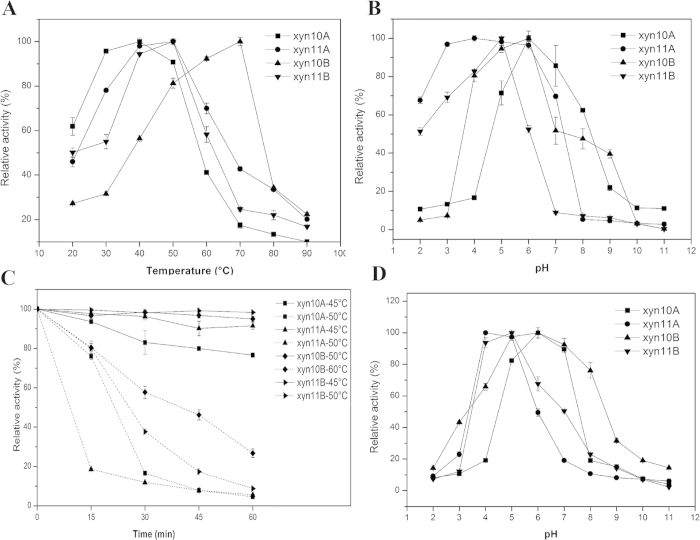
Optimal temperature (**A**), pH (**B**), thermostability (**C**) and pH stability (**D**) of purified recombinant xylanases. The optimal temperature for xylanase activity was determined by measuring activity at various temperatures using 1% beech wood xylan as a substrate. The influence of pH on enzyme activity was determined in various 50 mM acetate buffers using 1% beech wood xylan as a substrate. The data represent the mean of three replicates, and bars indicate the standard deviation of the three replicates. The thermal stability was evaluated as the relative residual activity after incubation without the substrate at various temperatures at optimal pH values. The pH stability was shown as the remaining activity after incubation for 30 min at the optimal temperature in buffers of various pH values without the substrate. The data represent the means of three replicates, and bars indicate the standard deviation of the three replicates.

The effects of temperature and pH on the thermostability of the four recombinant enzymes were investigated. The optimal temperatures of xyn10A, xyn11A, xyn10B and xyn11B for xylanase activity were 40, 50, 70 and 50 °C, respectively (Fig. 6A). The xyn10A, xyn11A, xyn10B and xyn11B enzymes displayed optimum activity at pH values of 6.0, 4.0, 6.0 and 5.0, respectively (Fig. 6B). An investigation of the thermal stability of the four recombinant enzymes showed that xyn10A, xyn11A and xyn11B were thermostable at 45 °C, and xyn10B was thermostable at 50 °C. After incubation at 50 °C for 30 min, the xylanase activity of xyn10A, xyn11A and xyn11B rapidly decreased, with a loss of approximately 80% (Fig. 6C). In contrast, xyn10B was considerably stable at the same temperature without a significant decline in activity (98% activity). The pH stability profiles showed that xyn10A and xyn10B were highly stable at pH values ranging from 5.0–7.0, and xyn11A and xyn11B were remarkably stable at pH values ranging from 4.0–5.0 (Fig. 6D).

#### Effect of metal ions and chemical reagents on enzyme activity

The effects of various metal ions (10 mM) and chemical reagents on the xylanase activity of the four recombinant xylanases were evaluated, and the results are shown in Table 2. The activities of the four xylanases were not substantially inhibited through Ca^2+^, Ba^2+^, Mg^2+^, Mn^2+^ and Li^+^, but were strongly inhibited through Hg^2+^, Cu^2+^ and Fe^3+^. The addition of DTT (1 mM), 0.1% Triton and 0.1% Tween-20 did not obviously influence the enzymatic activity of the four xylanases. In the presence of EDTA and β-mercaptoethanol (1 mM), the enzymatic activity of xyn11A was strongly inhibited, but the activities of xyn10A, xyn10B and xyn11B were only slightly inhibited. In the presence of 0.1% SDS, 72.3% of the activity of xyn10A, 23.9% of the activity of xyn11A, 73.2% of the activity of xyn10B and 3.5% of the activity of xyn11B remained.

#### Substrate specificity and kinetic parameters of various xylans

To obtain a better understanding of the functions and properties of each *P. oxalicum* GZ-2 xylanase, the specificity and kinetic parameters were investigated using several naturally occurring substrates, namely, oat spelt, birchwood and beechwood xylans. The enzymatic activities of the four xylanases were evaluated using various substrates to determine the enzyme specificity. The four xylanases were active toward polymeric xylans (oat spelt, birchwood and beechwood xylans) but not other substrates, such as CMC-Na, locust bean gum, konjac mannan and guar gum (Table S2). Although xyn10B degraded pNPX and pNPC, the other three enzymes were not active against these substrates.

The specific activity of xyn10A on beechwood xylan was significantly higher than that on birchwood and oat spelt xylans. For xyn11A, a higher specific activity for beechwood xylan or birchwood xylan than for oat spelt xylan was observed. The specific activities of xyn11B for oat spelt (602.7 ± 8.1 U/mg), birchwood (839.7 ± 20.1 U/mg) and beechwood (387.4 ± 11.1 U/mg) xylans were different. In contrast, xyn10B showed no difference in enzymatic specificity toward the three xylans (Table 3). The catalytic properties of the four enzymes were comparatively studied after determining the steady-state kinetic constants (*K*_m_, *V*_max_, kcat and kcat/*K*_m_) toward different substrates (Table 3). The four xylanases showed different *K*_m_ values for all substrates. A comparison the *K*_m_ of all xylanases for the three xylans revealed the maximum and minimum *K*_m_ values for xyn10B on beechwood xylan (1.0 ± 0.1 mg/mL) and xyn11B on oat spelt xylan (11.6 ± 1.2 mg/mL), respectively. The *V*_max_ values of xyn10B and xyn11B were significantly higher than those of xyn10A and xyn11A. The four xylanases exhibited significantly different values of *V*_max_ using the same substrate. Each enzyme exhibited various *V*_max_ values toward different substrates. The turnover numbers (kcat) of all enzymes showed similar properties to the *V*_max_. The highest value for kcat/*K*m was obtained for xyn10B, a moderate value was obtained for xyn11B, and the weakest values were obtained for xyn10A and xyn11A, depending on the substrate.

#### Comparative analysis of the products of xylan hydrolysis using the four enzymes

**Figure 7:**
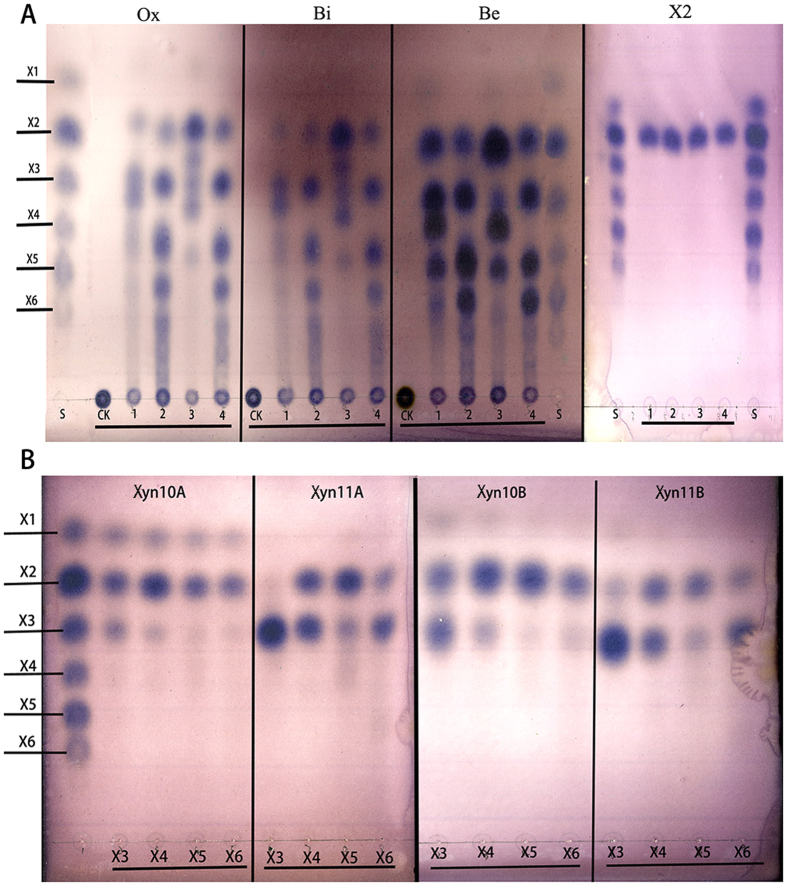
TLC analysis of the products after hydrolysis of various xylans (1%) and xylo-oligosaccharides (1 mg/ml) by purified recombinant xylanases. The purified recombinant enzyme and various substrates were incubated in 50 mM acetate buffer (pH 4.0) at 40 °C for 12 h. (**A**) The purified recombinant enzymes hydrolyzed Ox (oat spelt xylan), Bi (birchwood xylan), Be (beechwood xylan) and X2. The numbers 1, 2, 3 and 4 represent enzyme xyn10A, xyn11A, xyn10B and xyn11B, respectively. (**B**) The purified recombinant enzymes hydrolyze xylo-oligosaccharides (X3-X6). S: xylose and xylo-oligosaccharides were used as standards.

TLC was used to analyze the hydrolyzing actions of the four enzymes on various xylan sources and xylo-oligosaccharides. The four xylanases efficiently degraded different types of xylan polymers, such as oat spelt, birchwood and beechwood xylans. The hydrolytic product profiles produced by the four xylanases using oat spelt and birchwood xylan substrates were similar (Fig. 7A). In contrast, an obvious difference in the hydrolysis profiles was observed for each xylanase, regardless of the xylan source, and this difference was particularly notable between enzymes derived from GH10 (xyn10A and xyn10B) and GH11 (xyn11A and xyn11B) (Fig. 7A). The major unsubstituted product generated by xyn10A was xylotriose (X3), whereas xyn10B produced xylobiose (X2) without producing significant amounts of xylotriose (X3) when oat spelt and birchwood xylans were used as substrates. The beechwood xylan hydrolysate profile obtained with xyn10A consisted of xylobiose (X2), xylotriose (X3) and xylotetraose (X_4_). The hydrolytic products produced by GH11 xyn11A and xyn11B primarily consisted of xylo-oligosaccharides, namely xylobiose (X2), xylotriose (X3), xylotetraose (X4) and xylopentose (X5). However, the hydrolysis profiles of GH10 xyn10A and xyn10B not only contained identified xylo-oligosaccharides but also contained undetermined products with Rf values between those of the unsubstituted oligomers used as standards. Xylose was sparsely released through all enzymes during the degradation of the three xylans.

TLC analysis of the products produced from the hydrolysis of xylo-oligosaccharides through the four xylanases revealed different product profiles. The products of GH10 xylanase (xyn10A) hydrolysis of xylo-oligosaccharides (X3 to X6) primarily consisted of xylobiose (Fig. 7B) with a small amount of xylose. The hydrolysis profile obtained from the GH10 xylanase (xyn10B) hydrolysate was similar to that of xyn10A, with xylobiose as the main product. A different TLC profile was obtained for xyn11A, where xylotriose (X3) was not degraded and no xylose was observed compared with the xyn10A xylo-oligosaccharide hydrolysate. X4, X5 and X6 were hydrolyzed to X2 and X3 through xyn11A. The content of the hydrolysis products depends on the substrate. For example, the content of each X2 and X3 product was equal when X4 was used as the substrate, whereas a high content of X2 and low content of X3 was produced for X5, and more X3 and less X2 were produced for X6. The profile of the hydrolytic products obtained with GH11 xylanase (xyn11B) was similar to that of xyn11A, and the concentration of the hydrolytic products did not change. Although the two GH10 enzymes rapidly degraded X3, X4, X5 and X6, the two GH11 enzymes could not effectively hydrolyze X3 (Fig. 7B).

## Discussion

The production of multiple xylanases in microorganisms has evolved diverse enzymes with different physiological properties for more effective degradation of the complex plant biomass^20^,^21^. The presence of multiple xylanase isoforms in *P. oxalicum* GZ-2 was observed after 7 days of fermentation in previous studies using zymography^5^,^18^. The occurrence of a multiple xylanases has been observed in other fungi, but the extent of diversity xylanases based on the number of active xylanase bands varies among different strains. For example, Badhan *et al*. showed that in *Myceliophthora* sp. rice straw induced the maximum number (6) of xylanase isoforms, followed by wheat straw (4)^22^. Five xylanase isoforms were observed in *Penicillium purpurogenum* grown on oat spelt xylan^23^, and *Aspergillus fumigatus* Z5 secreted seven xylanase isoforms when grown on rice straw^24^. Herein, multiple xylanases were characterized using zymography and additional biochemical methods to explore the questions regarding the role and origin of multiple xylanases in microbes.

The active xylanase bands (8 bands) detected on the zymogram gel corresponded to 6 different genes, suggesting that multiple xylanases primarily are generated from numerous independent genes. This result reflects genetic redundancy between the multiple xylanases also present in other microorganisms. In the biomass-degrading model fungus *T. reesei* Rut-C30, 6 xylanases encoded by 6 different genes have been demonstrated^8^. In the genome of *Penicillium decumbens* 114-2, 10 putative xylanases were predicted, but none of these enzymes have been validated^25^. Multiple xylanase enzymes from diverse genetic backgrounds with multiple biochemical properties are important for degrading the complex heterogeneous xylan polymers in nature^26^. The genetic redundancy did not exclusively reflect the presence of multiple xylanases of *P. oxalicum* GZ-2, as two xylanase active bands originating from the same gene but with different molecular weights were observed, indicating that one active band did not signify one independent xylanase. The bands of b7 and b8 were identified as common proteins encoded by the same gene (putative endo-beta-1,4-xylanase), likely reflecting proteolysis or post-translational modifications. The production of multiple xylan-degrading enzymes of *P. oxalicum* GZ-2 primarily resulted from reduplicate xylanase genes, and proteolysis also generated multiple xylanase active bands. Badhan *et al*. examined the potential role for proteases in xylanase multiplicity, showing that the expression of multiple xylanases was not significantly affected after the addition of protease inhibitors^27^.

According to the area of the hydrolysis zone, the area of b8 was the largest among all xylanase active bands (Fig. 1B), suggesting that xyn11A was the most abundant protein among the multiple xylanases, and its corresponding gene (*xyn11A*) was transcribed at the highest level among the xylanase genes regardless of the substrate used for incubation (Fig. 5). This result suggested that the expression levels of xylanase are mainly controlled by the transcript level. Similar to other enzymes, biomass-degrading enzymes are primarily regulated at the transcript level^28^. In a previously study, we showed that Avicel induced weak xylanase activity in *P. oxalicum* GZ-2^5^. This activity was consistent with the results of xylanase genes showing low expression levels in response to Avicel induction. Xylans effectively induce xylanases. At 6 hours after the addition of Avicel to the xylan culture, the expression of *xyn10A* was significantly decreased, suggesting that Avicel or its derivatives repressed the expression of this gene. Conversely, the expression levels of the other three xylanase genes (*xyn11A*, *xyn10B and xyn11B*) were not changed, suggesting that these genes were not sensitive to Avicel. The transcript copies of *xyn11A*, *xyn10B* and *xyn11B* were twice that of *xyn10A* when *P. oxalicum* GZ-2 was grown in Avicel culture, suggesting that Avicel suppressed *xyn10A* expression compared with other genes. The addition of xylans to Avicel culture rapidly increased the transcript copy numbers of the four xylanase genes, suggesting that these genes are under the control of the same regulator.

The molecular weights of the four recombinant xylanases correspond to the calculated molecular mass of the mature peptide, suggesting that all recombinant enzymes are not glycosylated. This result is consistent with the prediction obtained using the NetNGlyc 1.0 Server (http://www.cbs.dtu.dk/services/NetNGlyc/), which showed no putative glycosylation sites in the four xylanase amino acid sequences, suggesting that glycosylation might not account for the multiple xylanases in *P. oxalicum* GZ-2.

The four xylanases, characterized in the present study, showed interesting differences in term of optimum temperatures and pH values, consistent with other fungal xylanases^29^,^30^,^31^. At low temperature of 30 °C, xyn10A exhibited more than 95% of its maximum activity, whereas only 40% activity remained for xyn10B, suggesting differences in the adaptability between xyn10A and xyn10B are beneficial for efficient degradation under various conditions^26^,^30^,^32^. The four recombinant xylanases exhibited differences in the activity against various types of xylans and no enzymatic activities were observed for other polysaccharides, suggesting that multiple xylanases are xylan-specific degrading enzymes. However, only xyn10B showed enzymatic activity against pPNX and pPNC, suggesting this enzyme has multiple enzymatic functions. Xylanase XYN10G5 from *Phialophora* sp.^33^ and XynA from *Penicillium chrysosporium*^34^ also showed enzymatic activity against pNPC. Many xylanases have been reported to possess both xylanase and cellulase activities^35^,^36^,^37^. The composition, degree of substitution, and solubility of particular xylans are important factors that influence enzyme-specific activity^9^,^38^. Beechwood xylans have few modifications and contain approximately 94% xylose, resulting in a compound with higher solubility and more enzyme access^39^. The four xylanases of strain GZ-2 showed the highest binding affinities on beechwood xylan suggesting that this material was the best substrate.

Differences in the hydrolytic products between the four recombinant xylanases have previously been reported, suggesting that all enzymes are unable to degrade xylotriose, and some xylanases minimally hydrolyze xylotriose^12^. This trend was observed in the present study (Fig. 7). The actions of xylanases on various xylans and xylo-oligosaccharides primarily depend on the properties and the glycoside hydrolase family of the enzyme^6^,^31^,^40^. Obviously, different features between GH10 and GH11 xylanases from *P. oxalicum* GZ-2 were expected. The hydrolysis of xylo-oligosaccharides was similar to the hydrolysis of xylans for the four xylanases, indicating that the hydrolytic products are primarily dependent on the internal characteristics of the enzymes. The diversity of the xylanase properties, including multi-biochemical characteristics, might improve the effective utilization of xylans that exhibit a more powerful adaptation in complex environments^26^.

In conclusion, the results of the present study showed that the multiplicity of xylanases primarily results from genetic redundancy and the role of proteases in the filamentous fungus *P. oxalicum* GZ-2, showing biochemical evidence that the four xylanases exhibited diverse properties and high catalytic efficiency toward various xylans and are potentially useful for biotechnological applications. The multiple xylanases exhibited high complementarity of enzymatic activities and specificities and were excreted into the extracellular environment as part of a xylanolytic enzyme system, potentially leading to the efficient degradation of complex hemicellulose.

## Materials and Methods

### Molecular manipulations

#### Cultivation, RNA isolation and cDNA synthesis of *P. oxalicum* GZ-2

The *P. oxalicum* GZ-2 used in the present study was deposited at the China General Microbiological Culture Collection Center (CGMCC 7527) and isolated and identified as previously described^5^. The cultivation of *P. oxalicum* GZ-2 to produce multiple xylanases, RNA isolation and cDNA synthesis were performed according to Liao *et al*. using corncob as an inducer^18^. Briefly, powdered mycelia was suspended in the TRIzol reagent (Invitrogen, Carlsbad, USA), and total RNA was isolated according to the manufacturer’s instructions. The synthesis of cDNA and the reverse transcriptase (RT) reactions were performed using the PrimeScript™ RT Reagent Kit with gDNA Eraser (Takara, RR047A, Dalian, China).

#### Isolation and analysis of xylanase sequences from *P. oxalicum* GZ-2

The genomic DNA of *P. oxalicum* GZ-2 was extracted as previously described^41^. Degenerate primers (Table S1) were used to amplify the core regions of the xylanase genes (*xyn10A*, *xyn10B*, and *xyn11B*) using the genomic DNA of strain GZ-2 as a template, designed using the iCODEHOP program (http://blocks.fhcrc.org/codehop.html) based on the multiple sequence alignment of homologous amino acid sequences using the ClustalW2 program (http://www.genome.jp/tools/clustalw/). The PCR products were cloned into the pMD-T19 vector for sequencing and BLAST analysis. The 5′ and 3′ flanking regions of the core region were obtained using self-formed adaptor PCR (SEFA-PCR), according to the protocol of Wang *et al*.^42^, and the primers are listed in Table S1 (supplementary). The sequences of the 5′-end and 3′-end of the PCR products were aligned to the appropriate size, sequenced and assembled to obtain the full-length gene according to Liao *et al*.^18^. The xylanase gene (*xyn11A*) was isolated from *P. oxalicum* GZ-2 and cloned as previously described^18^.

#### Quantification of xylanase gene expression profiles

The amounts of the four xylanase gene (*xyn10A*, *xyn11A*, *xyn10B*, and *xyn11B*) transcripts were determined from various carbon source cultures of *P. oxalicum* GZ-2 using real-time quantitative PCR (q-PCR). After *P. oxalicum* GZ-2 was precultured for 48 h in 100 mL basal mineral medium containing 1% Avicel (A) and 1% beech wood xylan (X), A was added to the X culture to generate a mixed culture (A + X, A:X = 2:1); whereas, X was added to the A culture to generate a mixture culture (X + A, A:X = 2:1). After 6 h of incubation, total RNA was extracted from the mycelia samples for q-PCR analysis. All q-PCR analyses were performed on an ABI 7500 real-time PCR machine (Applied Biosystems, Foster City, United States). The q-PCR analysis was performed using the primers listed in Table S1 (supplementary). The copy number of the gene expression was calculated using the standard curve of each gene as previously described^43^. The transcript number of the β-actin gene was quantified as an internal standard using the following primers: actin-F (CTCCATCCAGGCCGTTCTG) and actin-R (CATGAGGTAGTCGGTCAAGTCAC).

#### Construction of plasmids and strains

The coding sequences of the xylanase genes (*xyn10A*, *xyn11A*, *xyn10B*, and *xyn11B*) were amplified using the cDNA as a template and PrimeSTAR™ HS DNA Polymerase (Takara, Dalian, China) with the corresponding primers shown in Table S1. The forward and reverse primers introduced the *Eco*RI and *Xba*I restrictions sites at the 5′ and 3′ ends of all coding sequences, respectively. After digestion with *Eco*RI and *Xba*I, the DNA inserts were ligated into the pPICZαA vector downstream of the α-factor signal peptide sequence. Proper construction was confirmed through restriction digestion and DNA sequencing, and the products were designated as pPIC-xyn10A, pPIC-xyn11A, pPIC-xyn10B, and pPIC-xyn11B. The resulting recombinant expression plasmids (pPIC-xyn10A, pPIC-xyn11A, pPIC-xyn10B, and pPIC-xyn11B) were linearized with *Pme*I or *Sac*I (New England BioLabs, Beverly, MA, USA) for integration into the *Pichia pastoris* genome.

#### Expression and purification of recombinant protein

The recombinant plasmids (pPIC-xyn10A, pPIC-xyn11A, pPIC-xyn10B, and pPIC-xyn11B) were transformed into *P. pastoris* X-33 or GS115 through electroporation (Gene Pulser Xcell™ Electroporation System, Bio-Rad, Hercules, CA, USA) according to the manufacturer’s instructions. The transformants were plated onto YPDS plates (1% yeast extract, 2% tryptone, 2% dextrose, 1 M sorbitol, 100 μg/mL Zeocin, and 2% agar) and incubated at 30 °C for 2–3 days until colonies appeared. The *P. pastoris* transformants were isolated on YPDS plates containing increasing concentrations of Zeocin ranging from 1000 to 2000 μg/mL to select multicopy vector strain transformants. Twenty colonies from the 2000 μg/mL Zeocin YPDS plates were inoculated into 10 mL of YPM (1% yeast extract and 2% tryptone) medium containing 1% methanol in a 50-mL flask and cultured at 28 °C on a rotary incubator at 250 rpm to induce enzyme expression. The enzymatic activity of each supernatant was analyzed to determine the transformant with the best secretion yield for each enzyme. The transformants with the highest xylanase activity in a culture supernatant following 3–4 days of incubation were used for further fermentation in 1-L flasks following the method of Bai *et al*.^44^. The culture supernatants were recovered after 3–4 days of methanol induction through centrifugation (10 min, 5,000 × g), and the suspensions were concentrated using a 10-kDa molecular weight cut-off (MWCO) ultrafiltration membrane (Sartorius, Göttingen, Germany). The expressed 6 × His-tagged proteins were further purified using Ni-NTA Sepharose (Qiagen, Valencia, CA) according to the manufacturer’s instructions.

### Biochemical characteristics of multiple xylanases

#### Enzyme assays, SDS-PAGE and zymogram analysis

The standard assay for xylanase activity was performed at 50 °C for 10 min in 50 mM acetate buffer (pH 4.0) containing 1.0% (w/v) beechwood xylan as a substrate. The amount of reducing sugar released was determined using the 3,5-dinitrosalicylic acid (DNS) method^45^. One unit (U) of enzyme activity was the amount of enzyme required to release 1 μmol of reducing sugar per min under the assay conditions using xylose as a standard. The molecular weight of purified proteins was approximately determined through SDS-PAGE and zymogram analysis. The protein profiles analyzed through SDS-PAGE and zymography were conducted as previously described^18^. Briefly, SDS-PAGE was performed using a 12% (w/v) polyacrylamide gel with a 5% stacking gel using the Mini-Protean II system (BioRad). The protein bands were visualized after staining with Coomassie Brilliant Blue R-250. For the zymogram analysis, SDS-PAGE gels containing 0.1% beechwood xylan were stained with 0.1% (w/v) Congo red. To identify the proteins, the protein spots of interest were excised and in-gel digested with trypsin according to Liu *et al*.^24^. The digested proteins were identified using Bruker ultrafleXtreme MALDI-TOF-MS/MS (Bruker, Daltonics, Karlsruhe, Germany). We searched for the protein candidates using the proteome database of *P. decumbens* 114-2 downloaded from the NCBI database using Mascot (Matrix Science, London, UK). The following search parameters were used: taxonomy fungi, enzyme trypsin, up to one missed cleavage, carbamidomethylation of cysteines as a fixed modification, oxidation of methionine as a variable modification, peptide mass tolerance of 120 ppm, and MS/MS tolerance of 0.6 Da. The identification of the proteins was considered positive when the Mascot score was *p *< 0.05.

#### Effect of temperature, pH and various reagents on enzymatic activity

The effects of temperature, pH, and various reagents on the enzymatic activity or stability were measured under standard assay conditions according to the method of Liao *et al*.^18^. Briefly, the optimal pH values for xylanase activities were determined using the DNS assay with beechwood xylan (1%, w/v) in various buffer solutions, including glycine-HCl buffer (pH 2.0), acetate buffer (pH 3.0–6.0), sodium phosphate buffer (pH 7.0–8.0), and glycine-NaOH buffer (pH 9.0–11.0) at the optimal temperature corresponding to each enzyme (xyn10A at 40 °C, xyn11A at 50 °C, xyn10B at 70 °C, and xyn11B at 50 °C). The optimal temperatures were also determined using the same xylanase assay with beechwood xylan (1%, w/v) as a substrate and temperatures ranging from 20 °C to 90 °C at the optimal pH corresponding to each enzyme (xyn10A at pH 6.0, xyn11A at pH 4.0, xyn10B at pH 6.0, and xyn11B at pH 5.0). The thermal stabilities were determined at the optimal pH for each enzyme at 45–60 °C for up to 60 min. The pH stabilities were determined at the optimal temperature for each enzyme in various pH buffers as described above. The residual activity was measured under the standard assay conditions.

#### Substrate specificity and kinetic parameters

The substrate specificity of the purified recombinant enzymes was investigated at the optimal temperatures at a pH of 5.0 in the following substrates (1%, w/v): oat spelt xylan (Sigma, USA), birchwood xylan (Sigma, USA), beechwood xylan (Sigma, USA), sodium carboxymethyl cellulose (CMC-Na, Sigma, USA), locust bean gum (Aladdin, Shanghai, China), konjac mannan (Aladdin, Shanghai, China), and guar gum (Aladdin, Shanghai, China). The enzyme activity was quantified using the DNS method. The specific activities against four artificial substrates (10 mM) containing p-nitrophenyl-β-D-glucopyranoside (pNPG), p-nitrophenyl-α-L-arabinofuranoside (pNPA), p-nitrophenyl-β-D-xylopyranoside (pNPX), and p-nitrophenyl β-D-cellobioside (pNPC) were determined using the method of Parry *et al*.^46^. The kinetic constants of the four recombinant enzymes were determined after measuring the initial rates at various concentrations of various xylans (oat spelt xylan, birchwood xylan, and beechwood xylan, 1 to 20 mg/mL) at 50 °C at an optimal pH for 10 min. The Michaelis-Menten constant (*K*_*m*_) and the maximum velocity (*V*_*max*_) were calculated using Lineweaver-Burk plots.

#### Thin-layer chromatography (TLC) analysis of the hydrolytic products

The hydrolysis of oat spelt xylan, birchwood xylan, beechwood xylan (1%, w/v) and xylo-oligosaccharides (1 mg/mL) was performed at the optimal pH of each enzyme.

The reaction mixture was incubated for 12 h at 45 °C in a water bath, and subsequently, the enzyme reaction was terminated after boiling for 10 min and freezing. All products were freeze-dried and redissolved in a methanol (50%). A total of 6 μL of each aliquot was spotted onto a TLC plate (Qingdao Haiyang Chemical Plant, Qingdao, China), and the plates were developed with a solvent system consisting of chloroform-acetic acid-water (3:6:1, v/v). The plates were sprayed with a staining solution containing a 9:1 (v/v) mixture of methanol and sulfuric acid with 0.2% orcinol and heated at 85 °C for 5–10 min. Xylose (X1) and xylo-oligosaccharides (Megazyme, Ireland) (xylobiose (X2), xylotriose (X3), xylotetraose (X4), xylopentaose (X5), and xylohexaose (X6)) were used as standards.

#### Bioinformatics analysis

The amino acid sequence similarity was analyzed using BLAST. Multiple sequence alignment was performed using the ClustalW2 program. Geneious software (http://www.geneious.com) was employed to assemble and analyze the DNA sequences. Putative signal peptides were predicted using SignalP (http://www.cbs.dtu.dk/services/SignalP/). The exon-intron structure of the full-length gene was predicted using the online software FGENESH (http://linux1.softberry.com/berry.phtml). The phylogenetic analysis was performed through the Neighbor-Joining method using the MEGA 5.0 program according to Koichiro *et al*.^47^. The bootstrap was set with 1000 replicates. The figure depicting the multiple sequence alignment was constructed using ESPript 3.0^48^. The accession numbers for the sequences encoding xyn10A, xyn11A, xyn10B, and xyn11B were KF233755, KF233756, KF233757, and KF233758, respectively.

## Additional Information

**How to cite this article**: Liao, H. *et al*. Functional diversity and properties of multiple xylanases from *Penicillium oxalicum* GZ-2. *Sci. Rep*. **5**, 12631; doi: 10.1038/srep12631 (2015).

## Supplementary Material

Supplementary Information

## Figures and Tables

**Table 1:** Zymogram active-bands identification analysis using MALDI-TOF MS/MS.

Denotation	Predicted protein function	Family	Signal Peptides	Protein score	Accession number
b1	putative endo-beta-1,4-xylanase	GH11	Y	55	525581488
b2	putative endo-beta-1,4-xylanase	GH30	Y	103	525578833
b3	putative endo-beta-1,4-xylanase	GH10	Y	143	525581225
b4	putative endo-beta-1,4-xylanase	GH10	Y	106	525586882
b5	putative endo-beta-1,4-xylanase	GH11	Y	129	525580908
b6	putative endo-beta-1,4-xylanase	GH11	Y	144	525580908
b7	putative endo-beta-1,4-xylanase	GH11	Y	173	525583278
b8	putative endo-beta-1,4-xylanase	GH11	Y	93	525583278

**Table 2:** Effect of metal ions and chemical reagents on the activity of the recombinant enzymes.

Chemicals	Relative activity (%)			
	xyn10A	xyn11A	xyn10B	xyn11B
Control	100.0± 0.4	100.0 ± 3.7	100.0 ± 1.6	100.0 ± 0.6
Ca^2+^	96.1 ± 0.8	118.9 ± 1.7	97.6 ± 0.7	100.1 ± 3.4
Cd^2+^	18.5 ± 1.0	116.2 ± 2.4	95.6 ± 0.7	78.9 ± 4.2
Cr^3+^	77.7 ± 2.8	110.9 ± 3.1	79.7 ± 0.8	79.4 ± 0.4
Co^2+^	73.6 ± 2.2	124.9 ± 2.5	37.2 ± 0.3	81.7 ± 1.6
Cu^2+^	3.6 ± 0.4	2.3 ± 0.16	3.9 ± 1.2	41.6 ± 2.6
Ba^2+^	95.7 ± 0.1	120.8 ± 2.6	105.1 ± 6.0	100.1 ± 2.4
Mg^2+^	93.9 ± 0.5	127.6 ± 2.0	94.4 ± 4.4	101.8 ± 2.7
Mn^2+^	91.6 ± 1.4	108.8 ± 2.8	98.3 ± 3.0	100.1 ± 2.7
Ni^2+^	77.1 ± 2.0	116.1 ± 2.5	93.9 ± 1.1	79.1 ± 3.1
Li^+^	85.1 ± 0.7	119.4 ± 2.3	93.7 ± 3.2	89.6 ± 3.9
Fe^2+^	88.4 ± 1.5	115.5 ± 3.1	146.4 ± 3.1	101.3 ± 2.5
Fe^3+^	0.8 ± 0.02	22.8 ± 1.0	8.7 ± 0.2	33.0 ± 0.5
Hg^2+^	6.3 ± 0.7	24.4 ± 2.5	0.0 ± 0.0	1.4 ± 0.4
1 mM β-Mercaptoethanol	99.2 ± 0.9	41.7 ± 0.1	98.5 ± 2.0	101.0 ± 5.3
1 mM EDTA	99.2 ± 1.0	7.0 ± 0.04	87.2 ± 1.5	79.7 ± 1.8
1 mM DTT	101.6 ± 1.4	100.2 ± 1.2	100.0 ± 1.3	98.3 ± 2.6
0.1% Triton	99.7 ± 1.6	100.0 ± 1.1	89.2 ± 1.0	88.8 ± 2.9
0.1% Tween-20	99.2 ± 1.6	100.8 ± 1.2	92.2 ± 2.0	104.6 ± 7.6
0.1% SDS	72.3 ± 9.1	23.9 ± 1.8	73.2 ± 4.8	3.5 ± 0.3

**Table 3:** Comparison of kinetic values of four recombinant xylanases with various xylans as substrate.

Enzyme	*Km* (mg/ml)			*Vmax*(U/mg)			kcat (1/s)			kcat/*Km* (ml/mg/s)		
	Ox	Bi	Be	Ox	Bi	Be	Ox	Bi	Be	Ox	Bi	Be
xyn10A	5.4 ± 0.4 Ac	3.3 ± 0.3 Bb	3.0 ± 0.3 Bb	181.3 ± 12.5 Bd	144.5± 8.1 Cc	247.4 ± 13.8 Ac	103.4 ± 7.1 Bc	82.3 ± 4.6 Cc	141.0 ± 7.9 Ac	19.2 ± 0.1 Ac	24.8 ± 0.6 Bc	47.2 ± 2.4 Ac
xyn11A	7.9 ± 0.8 Ab	2.8 ± 0.2 Bb	3.0 ±0.1 Bb	500.1 ± 52.5 Ac	115.2 ±13.7 Bc	244.8 ± 9.4 Bc	177.5 ± 18.6 Ac	40.9 ± 4.9 Cc	86.9 ± 3.4 Bc	22.4 ± 0.8 Bc	14.6 ± 0.6 Cc	28.7 ± 0.2 Ac
xyn10B	1.8 ± 0.0 Ad	1.3 ± 0.1 Bc	1.0 ± 0.1 Cc	1397.8 ± 38.2 Ab	1140.6 ± 30.4 Bb	914.4 ± 31 Cb	964.5 ± 26.4 Ab	787.0 ± 20.9 Bb	631.0 ± 21.4 Cb	535.5 ± 8.6 Ba	626.4 ± 17.9 Aa	648.1 ± 27.0 Aa
xyn11B	11.6 ± 1.2 Aa	7.5 ± 0.7 Ba	4.7 ± 0.8 Ca	4604.7 ± 469.6 Aa	3486.8 ± 276.1 Aa	4426.2 ± 397.0 Aa	2264.0 ± 230.9 Aa	1714.3 ± 135.8 Aa	2176.2 ± 195.2 Aa	195.9 ± 0.7 Bb	229.1 ± 2.1 Bb	467.8 ± 36.7 Ab

Ox: oat spelt xylan, Bi: birchwood xylan, Be: beechwood xylan. A, B, C: per substrate, values with the same letter in one row are not significantly different from each other (P-value <0.05).

a, b, c, d: per enzyme, values with the same letter in one column are not significantly different from each other (P-value <0.05).
