# Supplementary material for: Functional diversity and properties of multiple xylanases from Penicillium oxalicum GZ-2
Source: Sci Rep. 2015 Jul 30;5:12631. doi: 10.1038/srep12631 (PMC4519791; doi:10.1038/srep12631)
Supplement: Supplementary Information [file srep12631-s1.pdf]

Functional diversity and properties of multiple xylanases from *Penicillium oxalicum* GZ-2

Hanpeng Liao [1,2], Haiping Zheng [1], Shuixian Li [1], Zhong Wei [1], Xinlan Mei [1], Hongyu Ma [3], Qirong Shen [1] and Yangchun Xu*[1]

Hanpeng Liao (lhp91@126.com)

Haiping Zheng (260241395@.qq.com)

Shuixian Li (995151498@qq.com)

Zhong Wei (weizhong@njau.edu.cn)

Xinlan Mei (meixinlan@njau.edu.cn)

Hongyu Ma (mahongyu@njau.edu.cn)

Qirong Shen (shenqirong@njau.edu.cn)

Yangchun Xu (ycxu@njau.edu.cn)

[1] National Engineering Research Center for Organic-based Fertilizers, Jiangsu Collaborative Innovation Center for Solid Organic Waste Utilization, Nanjing Agricultural University, Nanjing, 210095, China

[2] Ningbo Urban Environment Observation and Research Station-NUEORS, Institute of Urban Environment, Chinese Academy of Sciences, No. 88 Zhong Ke Road, Ningbo 315830, China

[3] College of plant protection, Nanjing Agricultural University, Nanjing, 210095, China

*Corresponding author

Yangchun Xu

National Engineering Research Center for Organic-based Fertilizers, Jiangsu Collaborative Innovation Center for Solid Organic Waste Utilization, Nanjing Agricultural University, Nanjing, 210095, China

E-mail: ycxu@njau.edu.cn

Tel/Fax: +86 25 84396824

Table S1 Primers used in this study

Gene	Primers name	Primer purpose	Primer sequence	Size (bases)
	xyn10A-df	Degenerate primers	TCACCCCCGAGAACDSNATGAARTGG	26
	xyn10A-dr		GCCCCACACGGTGATNCCNAYRCA	24
	xyn10A-f	cDNA cloning primers	ATGGTTCAAATCAAGGCTGCCGCT	24
	xyn10A-r		TCAAGTACGTGTGCGTCTGAGC	22
	xyn10A-ef	Specific expression primers	CGGAATTCGCTCCTGCCGAGACTCTGGAGTCT	32
	xyn10A-er		GCTCTAGATACAGAGCGGCAGTGATGGCGTTGTAGGC	37
xyn10A	xyn10A-qf	Quantitative-PCR primers	CTTCGGTGGCTCTGACTA	18
	xyn10A-qr		TGGTGATGTGGTCCTTGA	18
	xyn10A-3-sp3	Amplify gene of the 3'-end by SEFA-PCR	TGTGTTAGTCGATTGNNNNNNNNNTCCCTC	30
	xyn10A-3-sp2		AAGAAGGGGAGACATACCGAGAGTG	25
	xyn10A-3-sp1		GGGAGTGCCAGACTGGAGAAGAGAG	25
	xyn10A-5-sp3	Amplify gene of the 5'-end by SEFA-PCR	CAGGGTATCCCCATCNNNNNNNNNGGTAAG	30
	xyn10A-5-sp2		TTAGCCTTGACGACGCCAACTACGG	25
	xyn10A-5-sp1		GTTCCCTGGGCAACAGCGTCTTCTA	25
	xyn11A-ef	Specific expression primers	CGGAATTC GCCCCTTCCACCGAGCA	25
xyn11A	xyn11A-er		GCTCTAGATAACCAGAGACCTGGACGCT	28
	xyn11A-qf	Quantitative-PCR primers	AACGGTGTGAACAATGACT	19
	xyn11A-qr		CCTGGTAGTTGAAGTTGCT	19
	xyn10B-df	Degenerate primers	TTCTACGCCTGGGACGTNGTNAAYGA	26
	xyn10B-dr		GCCCCAGACGGTGATNCCNAYRCA	24
	xyn11B-f	cDNA cloning primers	ATGGTTCATCTGTCTGCCACCTC	23
	xyn11B-r		TTACAAGCACTGAGAGTACCAGGGG	25
	xyn11B-ef	Specific expression primers	CGGAATTC GCCGGATTGAACGACGCTGCCAA	31
	xyn11B-er		GCTCTAGATACAAGCACTGAGAGTACCAGGGGT	33
xyn10B	xyn10B-qf	Quantitative-PCR primers	TCTCCAGGCTCACTTCAT	18
	xyn10B-qr		ATACCGACGCAGTTCTTG	18
	xyn11B-3-sp3	Amplify gene of the 3'-end by SEFA-PCR	CACGGAACTTGACATNNNNNNNNNCTTGCC	30
	xyn11B-3-sp2		GGTCTCCAGGCTCACTTCATCGTCG	25
	xyn11B-3-sp1		TTGTTCAGTCCTACGGTGCCGAGAT	25
	xyn11B-5-sp3	Amplify gene of the 5'-end by SEFA-PCR	CCAAACACTCTGGCGANNNNNNNNNGTCCTC	30
	xyn11B-5-sp2		CAGTGGCAAAGGCGATGGGGAGGTA	25
	xyn11B-5-sp1		CCGACGATGAAGTGAGCCTGGAGAC	25
	xyn11B-Bf	Degenerate primers	CAGACCGGCACGAAYAAYGGNTWYT	25
	xyn11B-Br		GCCGTAGTTCTCCATGATRTARTAYTCNA	29
	xyn11B-f	cDNA cloning primers	ATGATCTCTCTCTCCTCCGTGGCAAT	26
	xyn11B-r		CTACAGGCACTGGGAGTACCATTG	24
	xyn11B-ef	Specific expression primers	CGGAATTCCTTCCTAGCGACCAGTCTGTCAACCTCGCCG	39
	xyn11B-er		GCTCTAGATACAGGCACTGGGAGTACCATTGGTTAGAAG	39
xyn11B	xyn11B-qf	Quantitative-PCR primers	CGGTCAGTTCAGTGTCAA	18
	xyn11B-qr		CAGTAGTCCAGCCATAGATG	20
	xyn11B-3-sp3	Amplify gene of the 3'-end by SEFA-PCR	TCTCCACATGCCATTNNNNNNNNNTAATTC	30
	xyn11B-3-sp2		ATGACTGTGTCTGCCGGCAGCTCTT	25
	xyn11B-3-sp1		TATCTACAAGCACACGCAGGTCAAC	25
	xyn11B-5-sp3	Amplify gene of the 5'-end by SEFA-PCR	ATATTTCCATCTCGCNNNNNNNNNTGCCAT	30
	xyn11B-5-sp2		GATTCTTTCCGTCGAAGGCTACCAG	25
	xyn11B-5-sp1		ACCGTCACCACTGCCAACCACTTCA	25

V (G/C/A), B (G/C/T), D (G/A/T), N (A/T/C/G), Underline: Enzyme cutting site

Table S2 The specific activity of four enzymes toward various substrates (U/mg)

Substrate	xyn10A	xyn11A	xyn10B	xyn11B
Oat spelt xylan	85.3 ±0.6	103.9 ±3.5	891.1 ±13.6	602.7 ±8.1
Birchwood xylan	84.7 ±3.7	137.9 ±0.6	890.1 ±15.3	839.7 ±20.1
Beechwood xylan	119.6 ±0.8	132.3 ±1.4	877.6 ±18.6	387.4 ±11.1
CMC-Na	nd	nd	nd	nd
Locust bean gum	nd	nd	nd	nd
Konjac mannan	nd	nd	nd	nd
Guar gum	nd	nd	nd	nd
pNPX	nd	nd	4.1 ±0.14	nd
pNPA	nd	nd	nd	nd
pNPG	nd	nd	nd	nd
pNPC	nd	nd	0.5 ±0.04	nd

nd：Not detection
